# Syphilinum

**Published:** 1892-09

**Authors:** P. C. Sanderson

**Affiliations:** Philadelphia, Pa.


### SYPHILINUM $^{1M}$.

P. C. SANDERSON, M. D., PHILADELPHIA, PA.

Mr. P. W., æt. thirty, came to my office with following history: Five weeks previous was under treatment for chancre by old school physician. Chancre cauterized three different times. He became dissatisfied, and desired I should take his case. The chancre was assuming a phagadenic appearance. Secreting ichorous pus. Inguinal glands hard and indurated. Merc.-corr.$^{3x}$ four times a day for five weeks. No improvement. *Frænum of penis* eating away. Patient complaining. I felt discouraged. But upon reading Dr. Thomas Wilde's report of Syphilinum, HOMŒOPATHIC PHYSICIAN, July, 1891, concluded to test it.

Wrote to Dr. Swan for one thousandth potency; gave one dose a day at night for two weeks, when the chancre began to heal. Continued until the ulcer came to a standstill. I then gave a few doses of Psorinum$^{200}$ and wrote Dr. Swan for the DMM Syphilinum. Gave one dose a day. After eight months' treatment he is a well man. The disease eradicated, not suppressed. I would occasionally give a dose of Psorinum to make it work the Syphilinum.

This man is a mechanic and during this time working very hard. Otherwise I claim he might have been cured thirty days sooner. I should have mentioned a piece of absorbent cotton was laid upon the chancre to catch the discharge.

I am treating a lady with Syphilinum with wonderful results. Her disease contracted from vaccination when a child. As Dr. Wilde says, a holy show. She was under old school treatment twelve years.

CHANCROIDS.

CASE II.—Mr. W. L., æt. twenty-five. Penis terribly swollen; four ulcers. Could not retract prepuce. Also had gonorrhœa. To use the expression of the patient when looking at his penis, *The worst I ever seen.*

Treatment: Bathed penis in hot water until he was able to retract his prepuce; dusted Iodoform on ulcers to keep surfaces apart; gave Merc.-corr.$^{3x}$. Ulcers healed rapidly; changed to *Can-sat.* for the gonorrhœa. Cure was complete in about six weeks. I write this as a comparison to Case No. 1.

As I have eaten such wholesome food from THE HOMŒOPATHIC PHYSICIAN, I thought it my duty to give some in return.

